# Task-shifting and the recruitment and retention of eye care workers in under-served areas: a qualitative study of optometrists’ motivation in Ghana and Scotland

**DOI:** 10.1017/S1463423624000185

**Published:** 2024-05-31

**Authors:** Joel G. Somerville, Niall C. Strang, Sven Jonuscheit

**Affiliations:** 1 Glasgow Caledonian University, Glasgow, UK; 2 University of the Highlands and Islands, Inverness, UK

**Keywords:** eye care, human resources for health, recruitment, retention, task-shifting

## Abstract

**Aim:**

To assist policy-makers in improving access to eye care in under-served areas by analysing the relationship between motivational factors affecting the uptake of task-shifting in eye care and the recruitment and retention of optometrists in remote and rural areas.

**Background:**

The World Health Organization recommends two key strategies in tackling preventable blindness in under-served areas: improving human resources for health and task-shifting. The relationship between task-shifting and recruitment and retention of eye care workers in under-served areas is unknown. Ghana and Scotland are two countries from different levels of economic development that have notably expanded the roles of optometrists and struggle with rural recruitment and retention.

**Methods:**

Motivation was explored through semi-structured interviews with 19 optometrists in Ghana and Scotland with experience in remote and rural practice. Framework analysis was used to analyse interviews, explore the relationship between task-shifting and recruitment and retention and create recommendations for policy.

**Findings:**

The main motivational considerations included altruism, quality of life, learning and career opportunities, fulfilling potential, remuneration, stress of decision-making and collaboration. Motivational and demotivational factors for task-shifting and recruitment/retention shared many similar aspects.

**Discussion:**

Recruitment and retention in remote and rural areas require staff be incentivised to take up those positions, motivated to remain and given the adequate resources for personal and professional fulfilment. Task-shifting also requires incentivisation, motivation to continue and the resources to be productive. Many motivational factors influencing recruitment/retention and task-shifting are similar suggesting these two strategies can be compatible and complementary in improving access to eye care, although some factors are culture and context specific. Understanding optometrists’ motivation can help policy-makers improve rural recruitment and retention and plan services.

## Introduction

Blindness affects national healthcare resources and national economy in both high- and low-income countries (Eckert *et al*., 2015; Green *et al*., 2016; Pezzullo *et al*., 2018), and Burton *et al*. (2021) argue that blindness is a development issue affecting a country’s ability to achieve the Sustainable Development Goals. This disadvantages low-income countries and rural areas within both high- and low-income countries (Dussault and Franceschini, 2006; World Health Organization, 2016; Burton *et al*., 2021). The Vision 2020 campaign (World Health Organization, 2001) set out several strategic goals to improve access to eye care in these under-served areas including the development of human resources for health (McGavin, 1999) and task-shifting. Recruitment and retention of eye care workers can improve rural human resources for eye health (HReH) and subsequently improve access to eye care (World Health Organization, 2016). Task-shifting, the redistribution of tasks from one professional group to another, can also improve access to health care and equity in delivery (World Health Organization *et al*., 2007) and has been successfully used in eye care (Needle *et al*., 2008; Levy and Booth, 2015; Harper *et al*., 2016).

Task-shifting in eye care primarily refers to the expansion in the scope of practice of mid-level providers, such as optometrists, to cover tasks traditionally performed by ophthalmologists. There is a wide variety of mid-level eye care professionals worldwide, and the variety in nomenclature and legal remit can make comparison difficult (du Toit and Brian, 2009). The World Council of Optometry considers therapeutic prescribing to be the major characteristic of an optometrist performing an extended, task-shifted role, and there are relatively few countries worldwide that reach this criterion (World Council of Optometry, 2015). According to best estimates, the only countries with substantial numbers (> 100) of therapeutic prescribing optometrists are Australia, New Zealand, Colombia, Canada, the United States of America, China, Nepal, Pakistan, England, Scotland, Ghana, Ethiopia and Nigeria (Ovenseri-Ogbomo *et al*., 2011; Young, 2022; Naidoo *et al*., 2023).

A recent literature review (Somerville *et al*., 2024) demonstrated factors affecting successful task-shifting and factors affecting successful recruitment and/or retention of health workers in under-served areas are similar. Staff must be adequately trained and incentivised to take up positions in areas which are under-served, motivated to remain there and given the means of productivity for personal and professional fulfilment. Task-shifting also requires incentivisation to undertake the extra tasks, motivation to continue and the resources to be productive. Motivation is the underlying factor that drives recruitment, retention and task-shifting in health care in under-served areas (Somerville *et al*., 2024). It is unknown whether the individual motivational factors that incentivise recruitment and retention of the rural eye care workforce are compatible or complementary with the individual motivational factors that influence eye care workers to adopt a task-shifted role and remain in it.

A number of studies have assessed the recruitment and retention of physicians in rural areas of a range of high- and low-income contexts, including meta-analysis (Lee and Nichols, 2014; Holloway *et al*., 2020; Russell *et al*., 2021). Single studies exist assessing the recruitment and retention of optometrists in rural areas (Mashige *et al*., 2015; Ramson *et al*., 2016; Boadi-Kusi *et al*., 2018). However, none have investigated the impact of task-shifting on the motivation to work in a rural area. This study aims to investigate specifically the relationship between task-shifting (defined as optometrist therapeutic prescribing traditionally performed by ophthalmologists) and the motivation to work in a rural area by comparing and contrasting primary data from a high- and low-income context.

Ghana and Scotland are two countries from different levels of economic development that have notably expanded the roles of mid-level eye care providers (optometrists), allowing for training in use of therapeutic drugs and shared care ophthalmology (Needle *et al*., 2008; Ovenseri-Ogbomo *et al*., 2011; Jarvis and Ker, 2014; Jonuscheit *et al*. 2019; Jonuscheit *et al*., 2021; El-Abiary *et al*., 2021). The first graduates of the new six-year Doctor of Optometry programme in Ghana in 2008 allowed optometrists to prescribe medications historically only available to physicians (Oduntan *et al*., 2014). In Scotland, full therapeutic prescribing requires an additional qualification, available since 2008, on top of a four-year bachelor’s degree (The College of Optometrists, 2023) although all optometrists have had limited access to therapeutic drugs since 2004 (Scottish Government, 2017). Ghana and Scotland were chosen as a high- and low-income comparison as they have an optometry education system that historically shared similar curricula and therefore are similar in their extent of task-shifting (Ovenseri-Ogbomo *et al*., 2011; Oduntan *et al*., 2014; Jonuscheit *et al*., 2019), both countries implemented task-shifting in eye care in the form of non-medical prescribing in the same year (2008) and therefore have had similar timescales for the potential impact of these policies (Oduntan *et al*., 2014; Jonuscheit *et al*., 2019) and importantly both countries have reported difficulties in recruiting and retaining optometrists in remote and rural areas (Moll *et al*., 1994; Dickey *et al*., 2012; Chua *et al*., 2013; Ilechie *et al*., 2013).

## Aim

The aim of this qualitative study was to improve access to eye care in under-served areas by analysing the relationship between motivational factors affecting the uptake of task-shifting in eye care and motivational factors affecting the recruitment and retention of optometrists in remote and rural areas of Ghana and Scotland. By comparing the motivational factors for these two strategies of improving access to rural eye care (encouraging task-shifting and encouraging rural practice) comparisons could be made to assess whether these strategies are compatible.

## Methods

A qualitative, phenomenological design was used to approach this topic using individual interviews to explore the lived experiences of optometrists who have worked in rural areas of Ghana and Scotland. Optometrists with any experience of rural practice were eligible for participation. Rural areas in Ghana were considered as any region outside the Greater Accra and Ashanti regions. Rural areas of Scotland were considered as the Highlands and Islands region. A purposive sample of participants was used to gain a wide range of experiences, and potential participants were contacted by email via professional networking, social media advertisement and snowballing. Semi-structured, in-depth interviews with 19 optometrists were conducted: 10 in Scotland and 9 in Ghana, at which point saturation was reached. These were conducted online due to the coronavirus disease 2019 pandemic by the primary author, a doctoral student and optometrist working in Scotland with experience in eye care in West Africa and using an interview guide. After giving informed consent, participants were asked about their personal experiences of working as an optometrist in a rural area and their experiences of task-shifting. Seventeen hours of interviews were recorded and transcribed, and numbers were assigned to each participant for anonymity. Framework analysis, as described by Ritchie and Spencer (1994) and Somerville *et al*. (2023), with the aid of NVivo (QSR International Ltd, 2021) was chosen to analyse interviews. Framework analysis was chosen due to its transparency, logical step-wise procedure, repeatability, suitability for dealing with *a priori* issues and use for creating policy recommendations (Somerville *et al*., 2023). Ethical approval was obtained prior to commencement (reference number HLSLSA19072).

## Results

Framework analysis of in-depth interviews led to the development of key themes and subthemes (Table 1) which will be described in turn. Each theme was considered in terms of its relationship to living and working in a remote or rural area and its relationship to task-shifting.

**Table 1.** Table of key themes and sub-themes

Key themes	Sub-themes
Business	Rural impact on business
	Scope of practice impact on business
Collaboration and teamwork	Inter-professional relationships
	Relationship to government or health board
	Relationship to management
	Relationship to ophthalmologists and secondary care
	Training other
Family and relationships	Family and relationships
	Home and rural upbringing
	Relationship issues
	Relationships with patients
Fulfilment and motivation	Being fully utilised
	Helping others
	Faith
	Respect and appreciation
	Work–life balance
Money	Salary and remuneration
	Financial incentives
	Financial security
	Out-of-pocket expenses
Rural living	Preconceptions
	Accommodation and practicalities
	Quality of life
	Isolation
Rural working	Nature of rural working Rural complex patients and distance to care Interesting and unusual cases
	Working in isolation Achieving CPD Making ‘big calls’ Being the only available person
	Improving eye care Community outreach Creating a learning environment
Scope of practice (SOP)	General perceptions of SOP Optometry training and contentment with SOP Task-shifting Awareness of capabilities
	Benefits of SOP
	Challenges of SOP
Technology	Positives of technology
	Lacking technology or equipment
Learning opportunities and development	

CPD = Continuous professional development.

### Business

In Ghana, optometrists in rural areas worked exclusively within the government system though some were able to find small amounts of private business. In Scotland, all optometrists were contracted by the National Health Service for eye examinations. Scottish participants’ main source of income was selling optical appliances; however, a great need was perceived in rural areas for diagnosis and management of disease. The increased scope of practice was therefore seen as an ‘opportunity’ to meet a visible need but not a profitable one.

You know there’s a lot of opportunities to really get something going. If you could make a living doing that – Participant 11, Scotland

As a result, participants often reduced their participation in task-shifting in order to meet business needs. An increased scope of practice also involved large set-up costs and investing in equipment.

### Collaboration and teamwork

Collaboration and teamwork impacted the motivation of optometrists to move to a rural area, their ability to practice effectively in a rural area, their motivation to undertake task-shifting and their ability to conduct task-shifting effectively. Working in a multidisciplinary team was a motivating factor to many as it made work interesting and diverse and conversely demotivating if the team they worked with was poor. Collaboration with government and local health boards were generally viewed poorly.

The number one thing is that I felt for at least a decade has been a struggle- In the Highlands and Islands- Is the level of support and development available from the local health board… there hasn’t been an appetite or willingness to support professional development of community optometrists – Participant 5, Scotland

In Ghana, there were some positive aspects to the relationship with the government as government positions were perceived to be more stable and offered a sense of security.

Participants who described positive perceptions of ophthalmologists were motivated by ophthalmologists who were supportive of learning and who ‘trusted’ optometrists by respecting their abilities and giving responsibility accordingly. A good relationship with ophthalmologists reduced the sense of isolation for those working in rural areas. However, ophthalmologists were also perceived to hamper patient care by ‘resisting’ the task-shifting of optometrists.

They seem not to recognise optometrists to be capable of handling eye conditions and that is demotivating…once you are paired with an ophthalmic nurse or an ophthalmologist you are virtually limited in your scope of practice even though you can do exactly what they are doing. They think they are supposed to handle the medical aspect – Participant 14, Ghana

That’s where the problems lay when I was trying to do (the independent prescribing qualification), was they… they didn’t have very much respect for us. You know, as far as they were concerned, we were money-grabbing spec sellers – Participant 10, Scotland

Working in rural areas often meant connecting remotely to secondary care via technology, and this support was vital for successful rural eye care. These remote interactions were more likely to lead to a poor relationship.

Ghanaian participants mainly viewed hospital management in a negative light and would consider the quality of support from management as a critical motivational factor in choosing a job or considering a career move. In Scotland, the pressure from corporate management to be profitable caused stress for optometrists. In Scotland, employers that were ‘loyal and supportive’ (Participant 4, Scotland) were a motivating factor but were often viewed as creating commercial pressure on one end that was mutually exclusive to providing the best clinical care.

### Family and relationships

Family and relationships were important motivational factors for moving to or continuing to live and work in a rural area but did not have a direct impact on the motivation to undertake task-shifting or the ability to perform task-shifting. The availability of appropriate work in a rural area for spouses or good local schools was a significant motivating factor in choosing the location of a job. This was often framed around the idea of ‘development’ and that the optometrist is not only motivated by their own development but also the development opportunities for their family.

I would have to think about (my family) because they would need the chance to develop. And moving them into the rural area, if it was going to prevent them from development or the chance to have a job… – Participant 15, Ghana

For many optometrists, a sense of ‘home’ drew them towards a particular location. This may be where they were brought up or where they came to view ‘home’ through forging new relationships. The ability to find a spouse or maintain romantic relationships was also an important guiding factor in decision-making.

Good relationships with patients were also motivating to optometrists.

### Fulfilment and motivation

Being fully utilised or fulfilling potential was a major motivating factor for optometrists. Optometrists were frustrated and demotivated when they perceived themselves as being underutilised and were motivated when they experienced the opportunity to practice their full range of skills. In Ghana, participants who had experience working in urban areas often felt frustrated that they were limited to refraction and not fulfilling their potential. Rural areas in Ghana were seen as places where optometrists had a freer rein to practice their skills, and therefore, they became more attractive places to work despite the other difficulties.

I felt I was underused… basically I was just limited to refraction. And I didn’t feel that I was giving as much as I wanted to. So I moved… at least I could actually do more than just being in my comfort zone. So that more of less informed my decision to move to a rural area – Participant 15, Ghana

Altruism was the reason many optometrists chose their career, the reason they moved to a rural area and the reason they stayed in their job even if there were poor pay and conditions:

I have always wanted to be in a space where I can contribute something to people who are deprived or those who are at the base of the pyramid. So that’s been a passion. And in (the rural area) or in the Ghana health service I would say that opportunity is there… I really had that motivation to be in a space where I could also extend some help to people – Participant 2, Ghana

Many optometrists specifically stated that helping others was more motivating than money or any financial aspect of their career. In Ghana, the driving force behind this altruism was often deep religious faith.

For Ghanaian participants, in particular, respect and appreciation for their professional status of ‘doctor’ were highly motivating.

Work–life balance was a significant motivating factor with optometrists willing to make decisions on where they worked and how much responsibility they undertook and would give up the potential of a better salary in order to achieve a good work–life balance. This was especially true for participants in Scotland where better work–life balance was perceived as being more available in rural areas. In Ghana, rural working was often associated with high demand and full diaries creating stress.

### Money

Finances were a source of motivation to take a job in a rural area, to work effectively in a rural area, to take up a task-shifting role and to perform the task-shifted role effectively. Obtaining a high salary was not a highly motivating factor for any participant, but it was extremely demotivating when remuneration was insufficient or absent. Ghanaian participants felt their salary did not reflect their years of training or their abilities, especially in comparison to other health professions working in the government system.

The amount of training that went into coming out as an optometrist in Ghana compared to the level that optometrists were placed in terms of salary really didn’t match up – Participant 15, Ghana

Remuneration from the National Health Service is the main source of income for Scottish optometry business owners but was generally viewed as poor. Although task-shifting provided more interest and variety in work, rural patients were perceived to present with more complex clinical conditions, and it was demotivating to optometrists not to be paid for the service they were providing.

It is great to be of service to the community and to provide a really essential healthcare service, but you have to get paid… you are being underpaid very often (in) what you are doing (for) the amount of time you are putting in and that is quite demoralising – Participant 11, Scotland

The perceived lack of sufficient remuneration was also one reason why some rural participants in Ghana did not pursue an increased scope of practice or did not practice to their fullest clinical potential.

Optometrists are not willing to go into specialisation because once you specialise it doesn’t put money on the table… looking at the training you receive and then the responsibility that is bestowed on you there should be proper salary given –Participant 19, Ghana

It was, however, noted that non-financial incentives such as accommodation and transport were often of greater value to participants than financial ones, and financial security was often more important than the highest available salary.

It was demotivating for Ghanaian participants when they were forced to pay from their own pocket for necessary supplies to do their job.

### Rural living

The benefits and challenges of rural life affected participants’ decision-making to move location and their ability to undertake effective task-shifting. Many Ghanaians were demotivated by difficulties finding adequate accommodation, unreliable internet service, poor roads, extreme weather, high cost of living and an unreliable water and electricity supply. In Scotland, despite some practical difficulties, most Scottish participants’ primary motivator for moving to a rural area was quality of life.

We were coming home to give our children the same opportunities that we had growing up. Which is skiing on the doorstep and hill climbing and a nice environment. A locality where you know everybody… I didn’t chase money. You know I could have earned an awful lot more money going in other directions, but I did chase quality of life… If you go after the money, you lose the quality of life – Participant 10, Scotland

### Rural working

Rural practices in Ghana tend to be served only by public sector facilities which often lacked funds and did not fully meet the needs of the population. However, public sector jobs were seen as more secure and therefore attractive with the ‘assurance of being in the system’ (Participant 1, Ghana). Public sector work was also seen as being more diverse with a wider range of patients needing a more diverse set of skills.

(In) the government system the only advantage is you are sure to see the cases you may not see in private practice – Participant 16, Ghana

Patients in rural areas in both countries were often viewed as being more complex, and task-shifting helped reduce stress in clinical decision-making. Participants in both countries also expressed satisfaction in seeing and treating interesting and unusual conditions that were less likely to be seen in urban areas as this led to variety in work, learning opportunities and professional development.

It’s… one word… Exciting!… The cases are not just limited to allergies. I mean we have retinopathies, maculopathies… certain cases I have seriously just seen in books… It has helped me to grow… – Participant 18, Ghana

You end up having to think outside the box a lot…the real pros about working here is that you do get a real mix of things, you know. You’ve got to manage a lot of things yourself…it keeps it a bit more exciting, doesn’t it?… I think certainly if I was working in a practice where I was just refraction, refraction, refraction all day long and there wasn’t any mix to it I would get slightly bored of it… now I’m much more confident… – Participant 12, Scotland

Despite this, the perception of being the only available person to make the call was a source of stress, although some relished the challenge.

An integral part of rural working for many optometrists was the idea of improving access to eye care in their area. The opportunity to participate in outreach programmes in Ghana or design services based on community needs was motivating to many.

### Scope of practice

Ghanaian and Scottish optometrists were generally content with their scope of practice. A wide scope of practice gave practitioners more independence, made clinical decision-making easier, was seen as beneficial in setting up innovative projects and provided optometrists with more confidence.

Having done the Independent Prescribing course… I have a huge amount of confidence and I’ve changed a lot of my management for a lot of patients… so it has already helped me. Like already just the confidence it has given me… I definitely didn’t expect it – Participant 13, Scotland

The benefits to patients were what most participants focused on at great length. They demonstrated how they were able to reduce distances patients needed to travel, reduce loss to follow-up and described the perception of ‘closing the loop’ between diagnosis and treatment. Ultimately a wider scope of practice helped optometrists reach their goal of reducing preventable blindness, despite recognising the fact that an increased scope of practice came with an increased workload.

What it means is the basic volume of work for optometrists is also going to increase. And though it is going to be a challenge, one way or another we are also going to meet the eye care needs of the populace… one way or another it is also going to reduce the burden of blindness – Participant 19, Ghana

The main benefit is patient care… the ease of patient care and benefit to the patients is the key factor… it really does centre around patient care and the more you can care for your patient in practice the better for the patient – Participant 9, Scotland

Many participants, however, described that the benefits of the increased scope of practice are not possible to realise without the resources necessary to support it. The absence of drugs, consumables and protocols was a source of challenge, as well as the lack of support from the local health board, local hospital or ophthalmologists.

### Technology

The availability of equipment and technology was a source of motivation to optometrists and affected their ability to work in rural areas and to undertake task-shifting. Telemedicine in particular was viewed as important for rural Scotland because it increased access to eye care, allowed patients to be managed in the community and helped to create accurate referrals as well as assist in peer learning through shared images. Technology was viewed as having tremendous potential to make task-shifting a success in rural areas although the cost of equipment was a source of worry. Conversely, a lack of technology or equipment in Ghana was seen as highly demotivating. One optometrist described the provision of equipment as a highly significant factor in their motivation whether to accept a rural posting or not:

If I had a job offer in a rural area, I think the first thing that I was going to consider was whether instrumentations in the set-up that I was going to use, the place actually meets the standard… nothing was (more of) a challenge to me than having the adequate instrument to be able to support the services that I gave… if somebody is posted into an empty room that is very, very quite discouraging – Participant 15, Ghana

What should motivate you is having the needed equipment to work with. I cannot attend to patients with virtually nothing in the health centre. – Participant 14, Ghana

### Learning opportunities and development

Opportunities for learning, personal development and career development were all areas that participants were highly motivated by. Learning was seen as an end in itself as well as a way to achieve career development and personal development.

I knew that in the field of optometry there is more to learn… I would go to any length to develop myself to help the best I could – Participant 15, Ghana

All Ghanaian respondents saw rural working as an ‘opportunity’ for all these types of development and therefore were motivated to consider these posts. Rural working, therefore, was a way to be fulfilled.

## Discussion

Comparison of findings from participant interviews in Ghana and Scotland were considered under these headings: motivation for recruitment, motivation for retention, training for task-shifting and the means of productivity for task-shifting (Table 2).

**Table 2.** Summary of main findings

Study aim	Ghana	Scotland
Motivation for recruitment	Likely to move to rural area due to: Forced placement Desire to work for government Enhanced learning experience Altruism	Likely to move to a rural area if: From a rural area Previous experience of a rural area Seeking outdoor lifestyle
Motivation for retention	Demotivated by Poor remuneration Out-of-pocket expenses Being underutilised/inability to fulfil potential Lack of respect or support Work overload	Demotivated by Lack of support from health board Lack of respect/collaboration from secondary care Stress of making big calls
	Motivated to stay by Altruism Learning opportunities Interesting/varied work Being fully utilised/fulfilling potential Respect Teamwork/collaboration Opportunity for community outreach	Motivated to stay by Altruism Quality of life/outdoors Sense of home Interesting/varied work
	Hygiene factors Adequate salary Ability to make friends/meet spouse Education for children	Hygiene factors Adequate salary Job for spouse
Task-shifting Training Means of productivity	Requires Collaboration with hospitals and ophthalmologists Appropriate and reliable equipment Access to medications Transport, accommodation, connectivity Appropriate scope of practice	Requires Collaboration with health board and secondary care Funding from government Availability of CPD Basic connectivity Good equipment/technology

CPD = Continuous professional development.

### Recruitment

Harnessing the motivation of health workers to move to rural areas is a key element of effective recruitment. Recruitment of eye care providers in rural areas of Ghana should not rely on forced placement. Practitioners who are motivated to move to rural locations willingly are more likely to be retained, so efforts should concentrate on challenging preconceptions. Learning new skills, like being involved in shared care schemes, is a highly motivating factor for optometrists and is in line with evidence from other health professions (Mbemba *et al*., 2016). Optometrists in Ghana also display an altruistic spirit, and using their skills to help others is a large motivating factor in moving to areas where need is located. This is similar to findings from nursing and other health professions where altruism is highlighted as a motivating factor for rural working (Javanparast *et al*., 2011; Smith *et al*., 2013; Mpembeni *et al*., 2015). This shows that optometrists often make career decisions based on higher motivational needs like a sense of fulfilment as well as on practical considerations such as job security.

In Scotland, optometrists tend to move to rural areas if they were originally from a rural area, had previous experience living in a rural area or were seeking the outdoor lifestyle. Outdoor lifestyle was also an important factor for health workers in Canada where financial incentives were less important than quality of life (Koebisch *et al*., 2020), but this may not be effective for workers from all cultures. Policy-makers should recognise optometrists often chose the location of their job based on family factors, like a sense of home or recreation opportunities. In both Ghana and Scotland, a positive perception of rural areas should be nurtured at an early stage and harnessed to improve rural recruitment, for example, through rural exposure at the student stage. Other systematic reviews have found experience in rural areas or training in rural areas as the strongest predictor for subsequent rural working (Mbemba *et al*., 2016; Ogden *et al*., 2020; Russell *et al*., 2021). However, there is also evidence that in some low-income African countries, students from rural areas use medical training to escape rural areas (Lewallen *et al*., 2012). Therefore, other rural retention incentives should be considered in a holistic approach and considered beyond a single professional group.

### Retention

Recruitment strategies alone are not enough to improve access to eye care in rural areas in the long term. Other evidence shows that recruitment should be incentivised but that retention incentives, not just recruitment incentives, are an important factor in creating a sustainable rural workforce (Buykx *et al*., 2010; World Health Organization, 2021). Creating an attractive rural work environment should begin with basic factors that prevent demotivation. In Ghana, leaving rural areas is often motivated by financial or educational opportunity, both for the optometrist and their family. It is highly demotivating when optometrists receive poor remuneration for their services. Rural optometrists are often the only eye care providers in their region and are therefore forced to take on a much larger responsibility than their urban peers for less pay, even to the point of providing consumables and medications from their own pocket. Optometrists in this study often compared themselves to other health professions and felt overlooked in terms of pay and respect. The importance of learning as a way to increase knowledge, respect, salary and personal fulfilment is a key criterion for motivating optometrists in rural areas and is similar to motivational factors found for other types of health workers (Mbemba *et al*., 2016). Ensuring the link is visible between learning and subsequent respect, financial reward and personal fulfilment are vital.

For Scottish optometrists, the main sources of dissatisfaction come from outside influences such as the local health board, local secondary care and other professionals. Optometrists are demotivated when they feel their profession or expertise is held back or disrespected. Optometrists are demotivated when health boards appear unwilling to set up shared care schemes that promote the profession and collaboration with secondary care. Practitioners also see the importance and benefit of good relationships with ophthalmologists, a theme found in other studies (Spillane *et al*., 2021). Optometrists are motivated when they perceive themselves to be in a dynamic, functioning, multidisciplinary environment but are demotivated when they perceive their expertise to be belittled. Policy-makers should seek opportunities for collaborative environments that increase mutual respect within the ophthalmic professions. The stress of decision-making was also a demotivating factor for optometrists, especially in more remote regions, and could be improved by fast and appropriate ophthalmological advice and support based on digital evidence. This could reduce hospital referrals, improve collaboration and reduce the stress of making big calls.

Salary was not highly motivating for optometrists but highly demotivating if inadequate for a perceived good standard of living or low in proportion to effort or responsibility. The financial remuneration in rural areas should be attractive enough to encourage people to be retained in what is perceived to be a more challenging environment. The highest motivational factor for retention was still altruism. Optometrists are often willing to compromise on issues such as remuneration, stress and working hours if they perceived that they were providing an important health service to their communities. However, optometrists motivated by altruism are also highly demotivated when they are underutilised or feel unable to effect change. The legal scope of practice should accurately represent the capabilities of optometrists and allow them to solve the problems they realistically encounter. Salary should increase to represent increased responsibility to enhance retention.

### Task-shifting: training and the means of productivity

Task-shifting cannot be carried out effectively without training from ophthalmologists and the means of productivity: appropriate and reliable equipment; access to medications; transport, accommodation and basic connectivity; and legal scope of practice that is appropriate for the type of cases actually encountered.

Training is a highly motivational factor for optometrists, especially in Ghana, who were shown to pursue training and learning opportunities at the expense of other factors. This may be because the inability to help others is as demotivating as the inspiration to help others is motivating. Adequate training requires the investment of local hospitals and collaboration of ophthalmologists. A regular review of the scope of practice of optometrists should be undertaken in both countries to ensure the legal scope of practice is proportionate to the increasing skills of optometrists.

There also needs to be a means of productivity available to optometrists to undertake task-shifting. This includes provision of appropriate, reliable and well-maintained equipment, the ability to purchase equipment at subsidised rates, provision of consumables and access to medications. This is similar to findings in other rural settings in low-income countries (Willis-Shattuck *et al*., 2008; Okoroafor *et al*., 2021). The main means of productivity driving optometrists is the ability to be financially sustainable when delivering enhanced services. A restructuring of remuneration is required to reflect the skills, responsibility, equipment and chair time that are needed to deliver task-shifting in rural areas of Scotland. This would both encourage rural optometrists to train in task-shifting and provide a viable and sustainable business model.

### Strengths and limitations

This project provided insight into motivation for task-shifting and recruitment/retention. Considering the high level of task-shifting carried out in Ghana and Scotland, this cannot be directly generalised to other contexts, especially those with lower levels of task-shifting. The definition of ‘under-served’ was problematic in this study, and this study used ‘rural’ as a proxy for ‘under-served’ as there are very little data that are detailed enough to comprehensively demonstrate which areas of a country are under-served in eye care. It would have been helpful to have a fuller understanding of the reasons why some urban optometrists lack the desire to move to rural areas.

### Conclusion

Task-shifting has the potential to be compatible and complementary with the recruitment and retention of optometrists in rural areas as they share similar motivation. Task-shifting can facilitate factors that motivate optometrists such as a good learning environment, job security, interesting and varied work, collaboration, fulfilling potential and helping those in need. Task-shifting opportunities are readily available and useful in rural settings. However, task-shifting is currently perceived to be poorly remunerated in relation to the training required and responsibilities carried, there is a perception of a lack of support from health boards or government, there is a perceived lack of respect for the profession, and it increases workload significantly without reward. Task-shifting also increases the stress of making ‘big calls’, that is, important clinical decisions. Factors conducive to quality personal and family life are important. In order for task-shifting to be compatible with retention of optometrists in rural areas, these issues must be addressed.

It is essential to understand personal motivation of optometrists in order to improve recruitment and retention of eye care workers in rural areas as well as designing task-shifting roles. Enhancing HReH and designing better task-shifting roles can increase the access to eye care in under-served areas. The results of this study can be transferred with care to other contexts as it has been shown that recruitment and retention to rural areas and task-shifting have the potential to be compatible within eye care systems. Motivation may vary between cultures, and this should be considered when designing health systems. The results of this study may also be useful for other health professions that struggle with access to care in under-served areas.
